# REDIportal: millions of novel A-to-I RNA editing events from thousands of RNAseq experiments

**DOI:** 10.1093/nar/gkaa916

**Published:** 2020-10-26

**Authors:** Luigi Mansi, Marco Antonio Tangaro, Claudio Lo Giudice, Tiziano Flati, Eli Kopel, Amos Avraham Schaffer, Tiziana Castrignanò, Giovanni Chillemi, Graziano Pesole, Ernesto Picardi

**Affiliations:** Department of Biosciences, Biotechnologies and Biopharmaceutics (DBBB), University of Bari, Via Orabona 4, 70125 Bari, Italy; Institute of Biomembranes, Bioenergetics and Molecular Biotechnologies (IBIOM), National Research Council, Via Amendola 122/O, 70126 Bari, Italy; Department of Biosciences, Biotechnologies and Biopharmaceutics (DBBB), University of Bari, Via Orabona 4, 70125 Bari, Italy; SCAI-Super Computing Applications and Innovation Department, CINECA, Via dei Tizii 6B, 00185 Rome, Italy; Mina and Everard Goodman Faculty of Life Sciences, Bar-Ilan University, 52900 Ramat Gan, Israel; Mina and Everard Goodman Faculty of Life Sciences, Bar-Ilan University, 52900 Ramat Gan, Israel; Department of Ecological and Biological Sciences (DEB), University of Tuscia, Via S. Camillo de Lellis 44, 01100 Viterbo, Italy; Department for Innovation in Biological, Agro-food and Forest systems (DIBAF), University of Tuscia, Via S. Camillo de Lellis 44, 01100 Viterbo, Italy; Department of Biosciences, Biotechnologies and Biopharmaceutics (DBBB), University of Bari, Via Orabona 4, 70125 Bari, Italy; Institute of Biomembranes, Bioenergetics and Molecular Biotechnologies (IBIOM), National Research Council, Via Amendola 122/O, 70126 Bari, Italy; National Institute of Biostructures and Biosystems (INBB), 00136 Roma, Italy; Department of Biosciences, Biotechnologies and Biopharmaceutics (DBBB), University of Bari, Via Orabona 4, 70125 Bari, Italy; Institute of Biomembranes, Bioenergetics and Molecular Biotechnologies (IBIOM), National Research Council, Via Amendola 122/O, 70126 Bari, Italy; National Institute of Biostructures and Biosystems (INBB), 00136 Roma, Italy

## Abstract

RNA editing is a relevant epitranscriptome phenomenon able to increase the transcriptome and proteome diversity of eukaryotic organisms. ADAR mediated RNA editing is widespread in humans in which millions of A-to-I changes modify thousands of primary transcripts. RNA editing has pivotal roles in the regulation of gene expression or modulation of the innate immune response or functioning of several neurotransmitter receptors. Massive transcriptome sequencing has fostered the research in this field. Nonetheless, different aspects of the RNA editing biology are still unknown and need to be elucidated. To support the study of A-to-I RNA editing we have updated our REDIportal catalogue raising its content to about 16 millions of events detected in 9642 human RNAseq samples from the GTEx project by using a dedicated pipeline based on the HPC version of the REDItools software. REDIportal now allows searches at sample level, provides overviews of RNA editing profiles per each RNAseq experiment, implements a Gene View module to look at individual events in their genic context and hosts the CLAIRE database. Starting from this novel version, REDIportal will start collecting non-human RNA editing changes for comparative genomics investigations. The database is freely available at http://srv00.recas.ba.infn.it/atlas/index.html.

## INTRODUCTION

RNA editing refers to a group of non-transient epitranscriptome modifications altering primary RNA transcripts through the insertion/deletion of specific nucleotides or base substitutions (1). The deamination of adenosine (A) in inosine (I) is the most common type of RNA editing, affecting thousands of nuclear and cytoplasmic transcripts in a variety of eukaryotic organisms (2). In humans, as well as in other mammals, the A-to-I conversion is mediated by members of the ADAR family of enzymes acting on double stranded (ds) RNAs which comprises ADAR (also known as ADAR1) and ADARB1 (also known as ADAR2), expressed in the majority of tissues, and ADARB2 (also known as ADAR3) found mainly in the nervous central system and thought to be catalytically inactive (3). A-to-I editing events are prominent in long double-stranded RNAs (dsRNAs) located in non-coding regions and formed by repeated elements in opposite orientation (mainly Alu sequences) (2). By contrast, the list of ADAR substrates in protein coding genes is relatively small (4).

Since Inosine mimics the properties of guanosine (G), it is commonly recognised as G by transcription and translation machineries (other than sequencing enzymes). As a consequence, A-to-I RNA editing can increase the transcriptome and proteome diversity, generate or destroy splice sites, alter codon identity or base-pairing interactions within higher-order RNA structures. Several evidences indicate that ADAR mediated RNA editing plays pivotal functional roles, tuning gene expression (5,6) or modulating the innate immune response through the MDA5-MAVS axis (2,7). Additionally, its deregulation is under active investigation being linked to different human disorders (8) including neurological (9,10), autoimmune (11), cardiovascular diseases (12) and cancer (13,14).

RNA editing has a great and promising therapeutic potential (15). Indeed, in contrast to CRISPR gene editing in which the phenotype rescue could be associated to undesired immune system responses (16) or accidental permanent genome changes (17), RNA editing could allow temporary fixes that eliminate candidate mutations with reduced adverse collateral effects (18,19).

The application of programmable A-to-I editing as well as the study of key events linked to human disorders or the investigation of still unknown RNA editing properties and functions or the development of advanced computing methods based on artificial intelligence require large and accurate collections of RNA editing events from a variety of transcriptome experiments. A few years ago we released REDIportal (20), a specialized database for A-to-I editing comprising >4.5 millions of events in 55 body sites of 150 healthy individuals from the Genotype-Tissue Expression (GTEx) project (https://gtexportal.org/home/). Currently, REDIportal is the unique and comprehensive resource for human A-to-I editing. Indeed, other competing databases such as RADAR (21) and DARNED (22) are outdated or in dismission.

REDIportal has been initially developed contrasting 2660 GTEx RNAseq experiments with a large collection of known A-to-I editing sites detected in six human tissues with the addiction of data from the RADAR database. Although the bioinformatic identification of A-to-I editing is easy, its profiling at single nucleotide level in a huge amount of RNAseq data is computationally intensive. Also, in a recent work dealing with the dynamic landscape and regulation of RNA editing in mammals, authors describe A-to-I editing in 8551 human GTEx RNAseq data but only 381 of these were really profiled at single nucleotide resolution (23).

To provide a comprehensive overview of RNA editing in humans, we describe here a novel release of REDIportal comprising about 16 millions of A-to-I events detected *de novo* at single nucleotide resolution in 9642 GTEx RNAseq data. Editing candidates have been identified using an *ad hoc* bioinformatics protocol based on our REDItools algorithm optimized for High Performance Computing (HPC) systems (24–26). The current version includes also events detected in hyper-edited reads that fail to be correctly aligned to the genome (27).

The novel REDIportal database stores individual A-to-I positions as well as statistics and relevant metrics per each GTEx sample, such as the Alu Editing Index (AEI) or the Recoding Editing Index (REI) or the expression of ADAR genes, that are expected to facilitate the RNA editing investigations. Now users can browse and visualize editing sites through our embedded and updated JBrowse or explore them in their genic context by our novel Gene View functionality.

With this release, REDIportal officially starts collecting RNA editing in non-human organisms, providing annotations for 107 094 A-to-I events from mouse nascent RNAseq data (5).

The REDIportal resource also includes the Cell Line A-to-I Rna Editing (CLAIRE) database (http://srv00.recas.ba.infn.it/atlas/claire.html) (28), making our portal a reference point for the scientific community and all researchers involved in the RNA editing field.

## DATA COLLECTION AND PROCESSING

### Data collection

**Figure 1.**
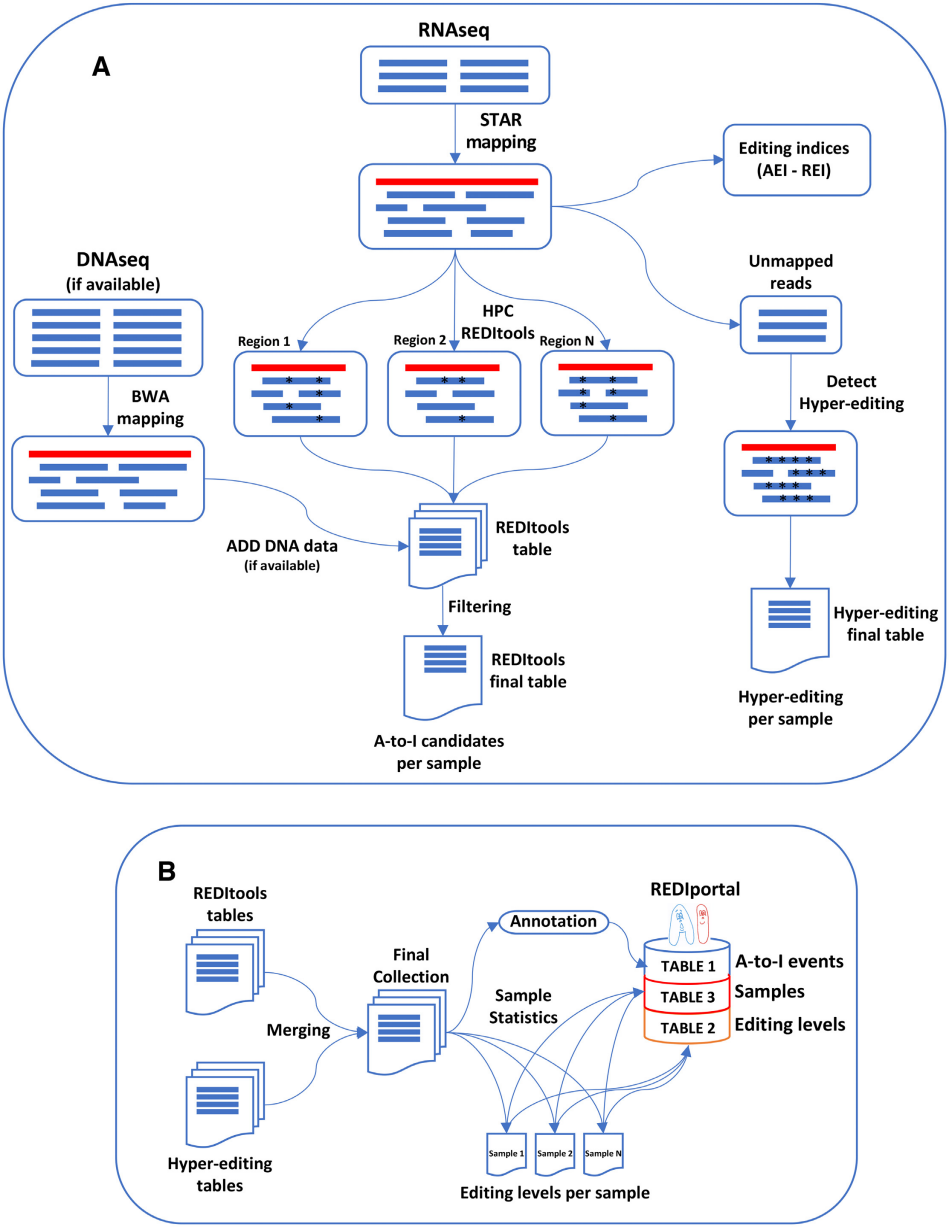
Data processing and database construction. (**A**) RNAseq data in fastq format are aligned on the human genome by STAR and converted in BAM files. In parallel, if DNAseq reads are available, are aligned on the same genome version by BWA. RNAseq BAM files are analyzed by HPC REDItools and the editing calling is distribute across different computing nodes, each working on a given genomic region. Resulting REDItools tables undergo to further filtering steps before the generation of the final table of A-to-I candidates. RNAseq unmapped reads are re-analyzed to detect hyper-edited reads and provide a list of hyper-editing sites per sample. RNAseq BAM files are further used to calculate the AEI and REI indices. (**B**) REDItools table and hyper-editing tables are merged in the final REDIportal collection. All events are annotated and stored in the MySQL TABLE1. They are also used to interrogate all RNAseq data to recover RNA editing levels and populate the MySQL TABLE2. Main RNA editing statistics per sample are also computed and collected in the MySQL TABLE3. Blue rectangles are reads, red rectangles are genomic regions, while black stars are A-to-I candidates.

We downloaded 9642 GTEx RNAseq experiments from the database of Genotypes and Phenotypes (dbGaP) (https://www.ncbi.nlm.nih.gov/gap/) (29) under the accession number phs000424.v7.p2 in sra format and converted them in standard fastq using the fastq-dump program of the SRA toolkit (http://ncbi.github.io/sra-tools/). In all cases in which WGS data (DNAseq) were available, they were downloaded from dbGaP in sra format and converted in raw fastq by fastq-dump. RNAseq reads were aligned onto the human genome (hg19/GRCh37) using STAR (version 2.5) (30) providing known gene annotations from Gencode (version 31) (31) (Figure 1A). DNAseq reads, instead, were aligned onto the human genome (hg19/GRCh37) using BWA (version 0.7) (32) (Figure 1A). All alignments were saved in sorted and indexed BAM files using SAMtools (version 1.9) (33).

### RNA editing detection

The *de novo* detection of RNA editing events was performed at the CINECA HPC Data Center (Italy) consuming ∼30 millions of CPU hours running an optimized version of our REDItools package whose algorithm scales almost linearly with the number of available cores (25). Indeed, the editing identification in massive transcriptome sequencing data is computationally intensive and time consuming, requiring the screening of the entire human genome, position by position, in order to look at nucleotide differences between RNA reads and the corresponding reference genomic site. In addition, depending on the sequencing throughput, individual genomic positions are supported by a very different number of RNA reads (sequencing depth), sometimes higher than 8000 counts. To speed up the browsing and traversing of aligned RNAseq reads, the editing detection was distributed over multiple computing nodes, each working on a given genomic interval. Moreover, the editing identification at nucleotide level was improved by a novel routine developed to increase the data loading efficiency, raising the algorithm performances of 8–10 times (25).

To identify all potential editing candidates the HPC version of REDItools was initially launched on individual BAM files of aligned RNAseq reads with non-stringent parameters. DNAseq support was subsequently added if available. Each REDItools table was then filtered according to our protocol described in Lo Giudice *et al.* (26) (Figure 1A). Briefly, all detected positions were annotated using known SNP sites, repeated elements in RepeatMasker and known editing events stored in the first release of the REDIportal database. SNPs and sites not supported by at least 10 DNAseq reads (if available) were removed, and the remaining positions were grouped in ALU (residing in Alu elements), REP NON ALU (residing in repetitive non Alu elements) and NON REP (residing in non-repetitive regions) groups, according to RepeatMasker annotations. While NON REP and REP NON ALU variants underwent a second round of REDItools using stringent call criteria to exclude multimapping reads and PCR duplicates, changes in ALU regions were filtered only by coverage (at least 5 reads) and base quality (phred score of at least 30). At the end, all filtered positions were collected returning the final list of RNA editing candidates per RNAseq (Figure 1A).

In parallel, unmapped reads per sample were analysed using the pipeline by Porath *et al.* (27) in order to identify A-to-I events in hyper-edited reads (Figure 1A).

### Annotation and downstream analyses

Filtered REDItools tables from 9642 samples were merged yielding 10 089 202 editing positions, while the union of hyper-edited tables returned 9 982 214 editing sites (Figure 1B). The merging between both collections yielded a comprehensive and non-redundant list of 15 683 855 *bona fide* A-to-I editing events. All positions were then annotated using ANNOVAR (34) (a standalone software to functionally annotate genetic variants) and the following updated databases: (i) RepeatMasker containing known repetitive elements; (ii) dbSNP (version 151) collecting genomic single nucleotide polymorphisms (35); (iii) Gencode (v34) (31), RefSeq (36) and UCSC (Genome Browser database) (37) storing gene and transcript annotations; (iv) PhastCons providing conservation scores across 100 species. Although RADAR and DARNED databases are not fully functional, known A-to-I changes from both repositories were added for continuity with the previous REDIportal database. The complete collection was also lifted to human genome hg38/GRCh38 by liftover (a free software to convert genome coordinates and genome annotation files between assemblies) and annotated using the corresponding hg38 databases. RADAR and DARNED positions were converted to hg38/GRCh38 accordingly.

Hg19 and hg38 annotated positions were finally loaded in the MySQL TABLE1 of REDIportal (Figure 1B).

All positions were also used to interrogate 9642 RNAseq samples to extract RNA editing levels as well as the number of reads supporting As and Gs per site. These values were collected in the MySQL TABLE2 of REDIportal (Figure 1B).

For each aligned RNAseq experiment, FeatureCounts (38) (a software for counting reads to genomic features such as genes, exons, promoters and genomic bins) was applied to count the number of reads per gene and a custom script was used to normalized these values in TPM (transcripts per million). Same aligned reads were also used as inputs to calculate the AEI and REI indices that are relevant metrics to measure the RNA editing activity, globally or at recoding sites, respectively (Figure 1A). The AEI was calculated using the RNAEditingIndexer program (https://github.com/a2iEditing/RNAEditingIndexer) (39), while the REI was computed as described in Silvestris *et al.* (13) and implemented in Lo Giudice *et al.* (40) (https://github.com/BioinfoUNIBA/QEdit).

The AEI was defined as the weighted average of editing events occurring in all adenosines within Alu elements, while the REI was the weighted average over all known recoding sites (i.e. residing in coding protein genes).

Finally, a custom script was used to collect main statistics for each GTEx sample and generate the MySQL TABLE3 of REDIportal (Figure 1B).

## DATABASE CONTENT AND WEB INTERFACE

### Database construction and content

As in the previous release, REDIportal allocates all 15 683 855 sites in two main MySQL tables. TABLE1 includes individual sites and their annotations, while TABLE2 stores RNA editing levels per RNAseq, tissue and body site. Statistics and RNA editing metrics per sample are instead stored in the novel TABLE3 MySQL table. It comprises the RNAseq run accession (according to dbGAP), the DNAseq run accession (if available), the organism name, the data source (GTEx or SRA), the body site and its status (healthy or diseased), the tissue type (bulk or single cell), the number of detected A-to-I events as well as the number of hyper-editing sites, the AEI and REI indices, and the expression of ADAR genes (ADAR, ADARB1 and ADARB2). TABLE3 contains also further statistics such as the distribution of editing sites over the three ALU, REP NON ALU and NON REP groups, the fraction of synonymous or non-synonymous events in protein coding regions, the distribution of editing sites across the gene structure, the fraction of edited and hyper-edited positions, and the distribution of RNA editing levels. In addition, it includes the fraction of edited and unedited genes, and the distribution of all variants detected per sample as a sort of quality check of the prediction.

REDIportal provides RNA editing details for 9642 RNAseq samples from 549 individuals across 31 tissues and 54 body sites. By means of the HPC REDItools based pipeline, each sample contains 60 873 edited events on average. The mean number of A-to-I events in hyper-edited reads per sample, instead, is 26 942. The highest number of events was detected in the cerebellar hemisphere and cerebellum (Supplementary Figure S1). By contrast, skeletal muscle and heart contained the lowest number of candidates. As already known by the literature, the majority of A-to-I events resides in Alu elements located mainly in intronic regions (Supplementary Figure S2) (41). A consistent fraction of sites was identified in intergenic and 3′ UTR regions (Supplementary Figure S3). A-to-I events in exonic regions, instead, were very limited (Supplementary Figure S3). The RNA editing activity, measured by the AEI index (39), was largely body site specific as well as the activity at recoding sites (Supplementary Figures S4 and S5).

### Web interface

The novel REDIportal inherits the layout from the previous version and all web pages are developed in Bootstrap, CSS and JavaScript. Server side operations to query MySQL tables and retrieve data are performed in Python (v2.7) and require MySQLdb (https://pypi.python.org/pypi/MySQL-python/1.2.5) and mxTextTools (http://www.egenix.com/products/python/mxBase/mxTextTools/) as external modules for MySQL connections and high-performance text manipulation, respectively.

**Figure 2.**
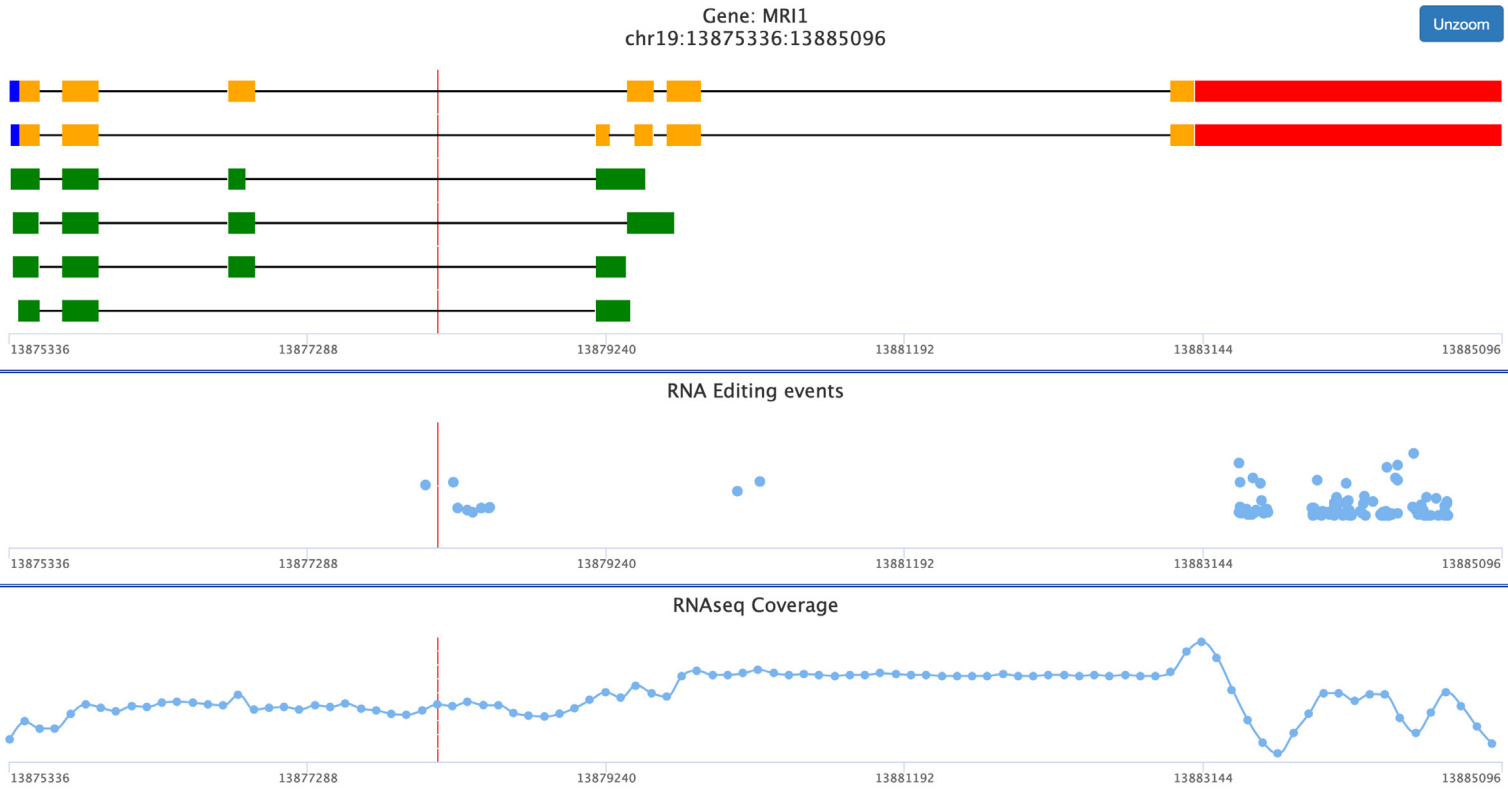
Example of Gene View page for gene MRI1. Gene View is a novel REDItools functionality in which users can visualize RNA editing events in their genic context. The web page shows at most three panels: (i) the gene structure with details about individual transcripts and specific features such as 5UTR for 5′ UTR in blue, 3UTR for 3′ UTR in red, CDS for the protein coding region in orange, Intron for intervening sequences in black and Exon for a non-coding exon in green; (ii) the list of RNA editing events in blue circles with related levels (the mean value is included in case of searches from the Gene View search page); (iii) the RNAseq coverage for the specific genomic region and related to the specific RNAseq experiment. This panel is visible only at sample level.

REDIportal now allows two main searches, at position level and sample level. Users can interrogate the database in the conventional way by providing a genomic region, in the format chr:start-end, or a specific gene symbol, or query the collection of samples by providing one or more run accessions and limiting results according to tunable AEI or ADAR expression values. The search dropdown menu now includes the Gene View page by which users can visualize RNA editing events in their genic context, zooming on specific gene regions if needed (Figure 2). Retrieved sites and samples are shown in dynamic and sortable tables automatically generated by DataTables in server-side mode to easily handle millions of rows.

**Figure 3.**
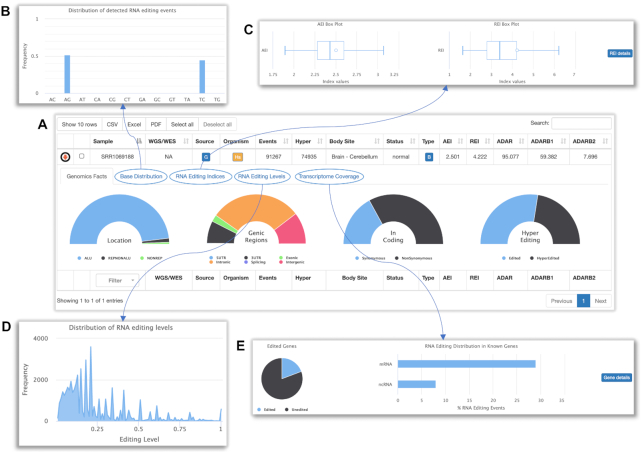
Example of sample search for run SRR1069188. The output of the sample search provides basic RNA editing info per RNAseq in a tabular format and enables the browsing of five panels with further details. (**A**) ‘Genomics Facts’ includes the location and distribution of detected sites in different genomic and genic regions; (**B**) ‘Base Distribution’ shows the graphical distribution of all detected variants by the REDItools based pipeline; (**C**) ‘RNA Editing Indices’ depicts box plots of AEI and REI indices calculated on the body site group of the retrieved sample. Specific index values are indicated in each plot by an empty circle; (**D**) ‘RNA Editing Levels’ shows the distribution of RNA editing levels; (**E**) ‘Transcriptome Coverage’ displays statistics about the fraction of edited and unedited genes, and the distribution of detected events in mRNAs or ncRNAs. Panels C and E include two additional buttons for further details about recoding events (Figure 3) and edited genes.

The sample search allows the browsing of main RNA editing statistics per sample and includes five panels (Figure 3): (i) ‘Genomics Facts’ with the location and distribution of detected sites in different genomic and genic regions; (ii) ‘Base Distribution’ with the graphical distribution of all detected variants (not limited to A-to-G or T-to-C); (iii) ‘RNA Editing Indices’ with box plots of AEI and REI indices calculated on the body site group of the retrieved sample; (iv) ‘RNA Editing Levels’ with the distribution of RNA editing levels; (v) ‘Transcriptome Coverage’ with statistics about the fraction of edited and unedited genes, and the distribution of detected events in mRNAs or ncRNAs.

**Figure 4.**
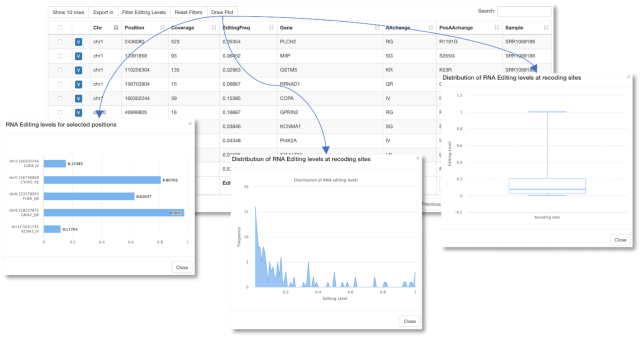
Details about recoding events. The ‘RNA Editing Indices’ panel comprises a button enabling the browsing of individual recoding events. Selected positions can be compared by a bar graph or all sites can be shown in dedicated plots.

Additionally, the ‘RNA Editing Indices’ panel as well as the ‘Transcriptome Coverage’ panel provide external buttons that enable the recovery of further details. The button ‘REI details’ allows the browsing and filtering of known RNA editing recoding events (Figure 4) and some visualization facilities for all or selected sites only. The button ‘Gene details’, instead, retrieves the list of edited genes per sample and enable the gene view for each gene.

RNA editing sites can also be retrieved by a dedicated Application Programming Interface (API). Differently from the web-browsing approach, API does not leverage as much bandwidth and can also be interfaced with third-party programs able to manage outputs in JSON format. API outputs can be displayed in the web-browser or in the commands shell.

## CONCLUSION AND FUTURE PLANS

RNA editing is emerging as a relevant epitranscriptome phenomenon involved in a variety of cellular functions. Although the massive transcriptome sequencing has accelerated the research in this field, different aspects of the RNA editing biology need to be elucidated. To facilitate the investigation of A-to-I RNA editing we have updated our just rich REDIportal catalogue distributing to the scientific community >15 millions of A-to-I changes in 9642 human RNAseq samples from the GTEx project. In contrast with the previous release in which RNA editing events were called from a known list of A-to-I changes from only six tissues (18 RNAseq experiments) (41), the version presented here includes editing candidates identified in all GTEx RNAseq data by applying a dedicated pipeline based on our HPC version of the REDItools software and consuming ∼30 million CPU hours through PRACE (Partnership for Advanced Computing in Europe) projects.

The current REDIportal release allows searches at sample level, provides overviews of RNA editing profiles per each RNAseq experiment, implements a Gene View module to look at individual events in their genic context, hosts the CLAIRE resource (28) and collects non-human RNA editing changes.

As a comprehensive catalogue of A-to-I RNA editing, REDIportal is in active development and thanks to two PRACE European projects for computing resources, its short-term goal will be the inclusion of A-to-I sites from all TCGA samples as well as events from RNAseq data produced in consortia for neurological and neurodegenerative disorders (CommonMind and PsychEncode) or RNAseq experiments from the Nonhuman Primate Reference Transcriptome Resource (NHPRTR) (42). Finally, REDIportal will be expanded with A-to-I changes detected in single cells.

## DATA AVAILABILITY

REDIportal is an open source database available through the web page http://srv00.recas.ba.infn.it/. REDItools is an open source software available in the GitHub repository (https://github.com/BioinfoUNIBA/REDItools).

## Supplementary Material

gkaa916_Supplemental_File
