# Supplementary material for: REDIportal: millions of novel A-to-I RNA editing events from thousands of RNAseq experiments
Source: Nucleic Acids Res. 2020 Oct 26;49(D1):D1012–9. doi: 10.1093/nar/gkaa916 (PMC7778987; doi:10.1093/nar/gkaa916)

Supplementary Figures

Figure S1. Distribution of REDIportal events per body site. In blue are events identified by the HPC REDItools pipeline, in orange, instead, are hyper editing sites.


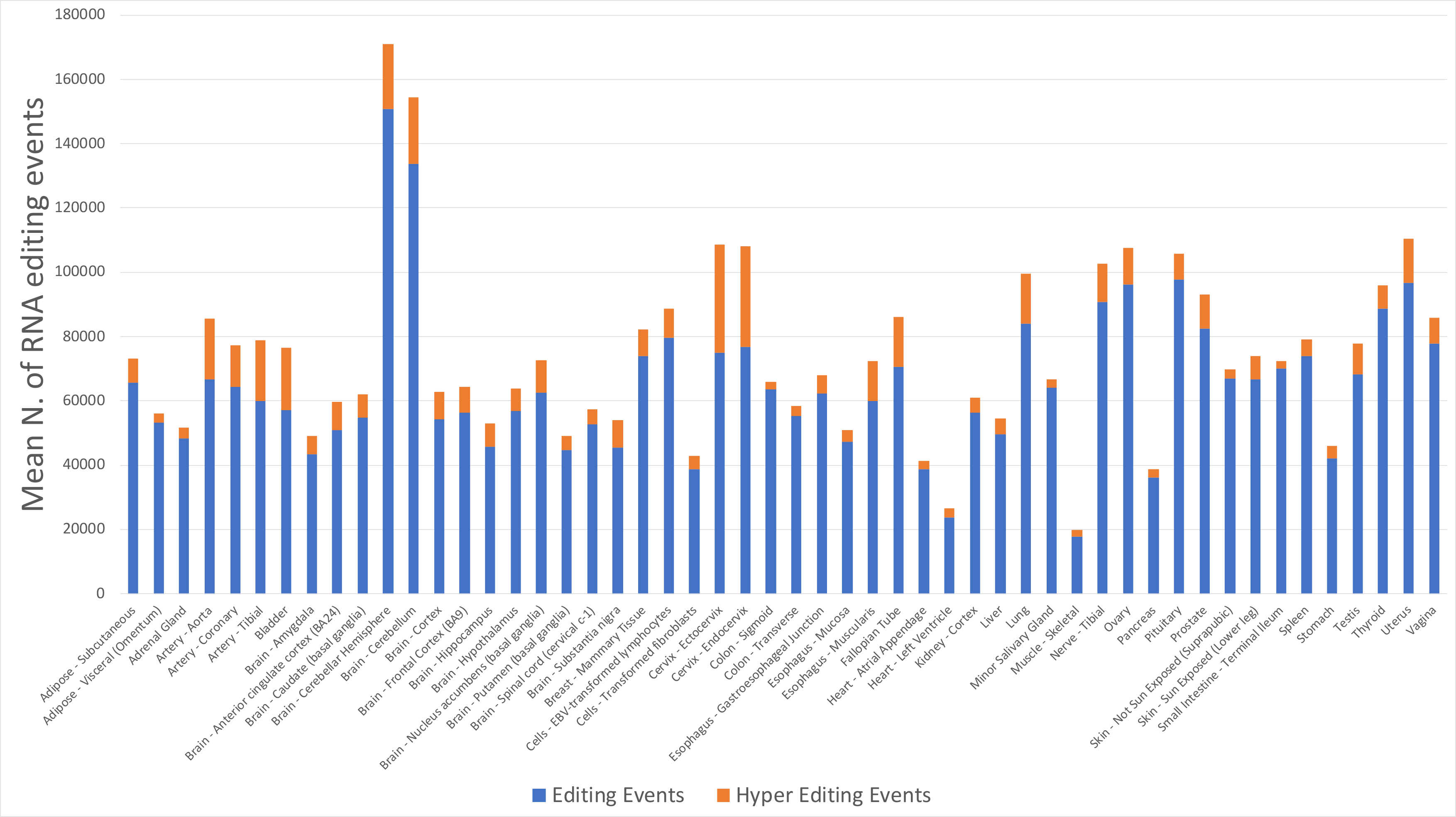


Figure S2. Distribution of REDIportal events per each ALU, REP NON ALU and NON REP group.


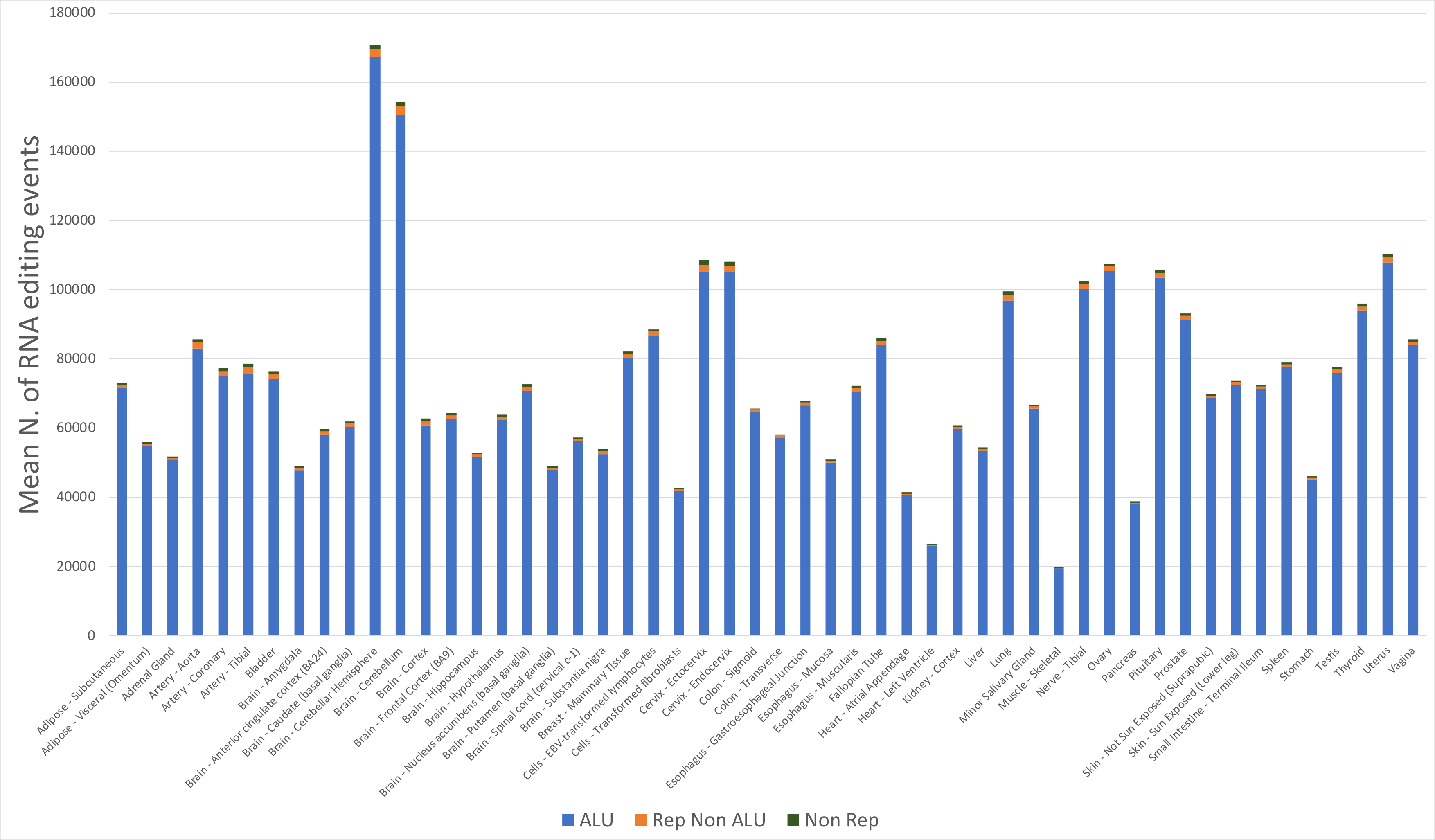


Figure S3. Distribution of REDIportal events per each gene region.


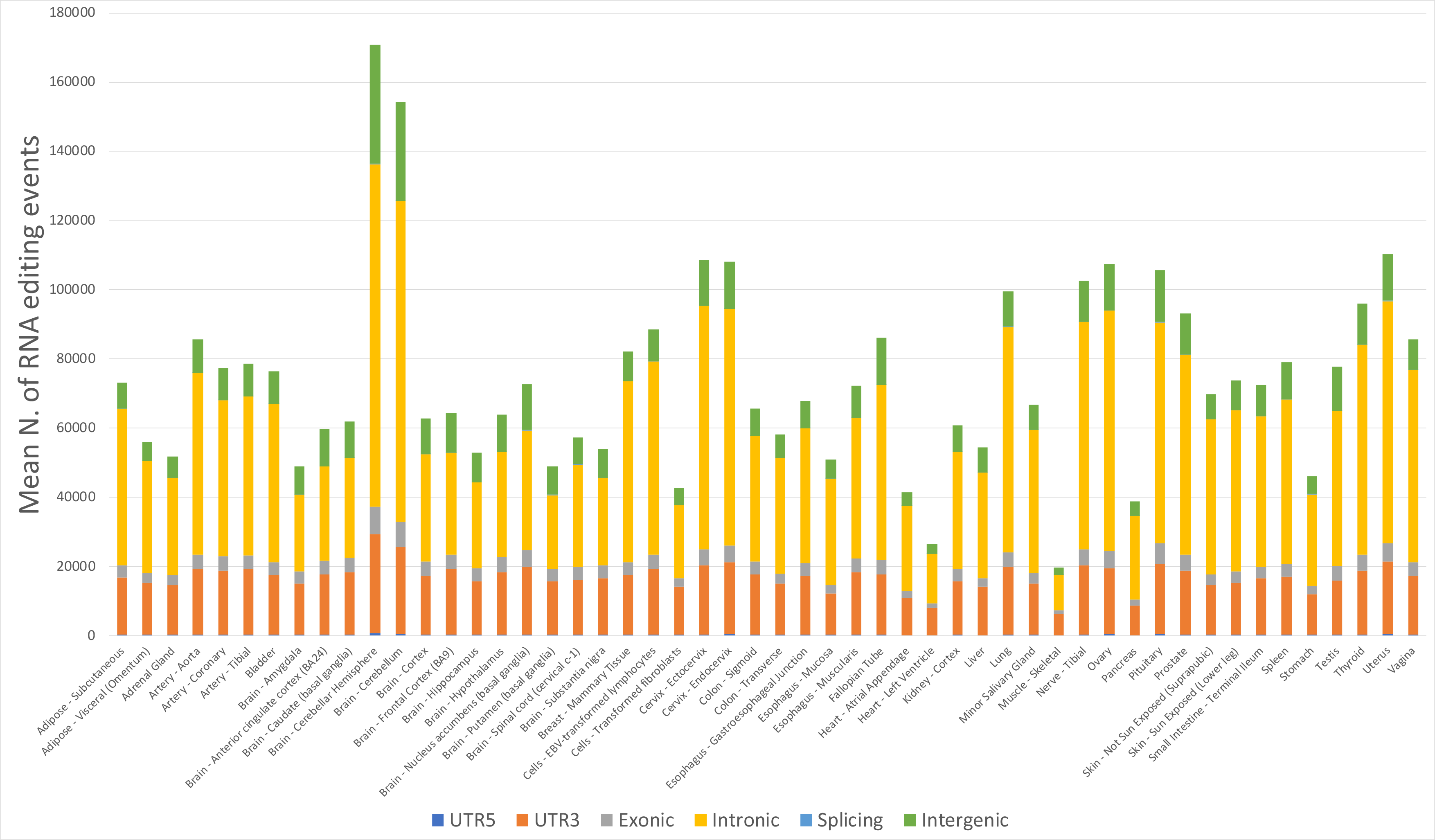


Figure S4. Distribution of AEI index values across body sites.


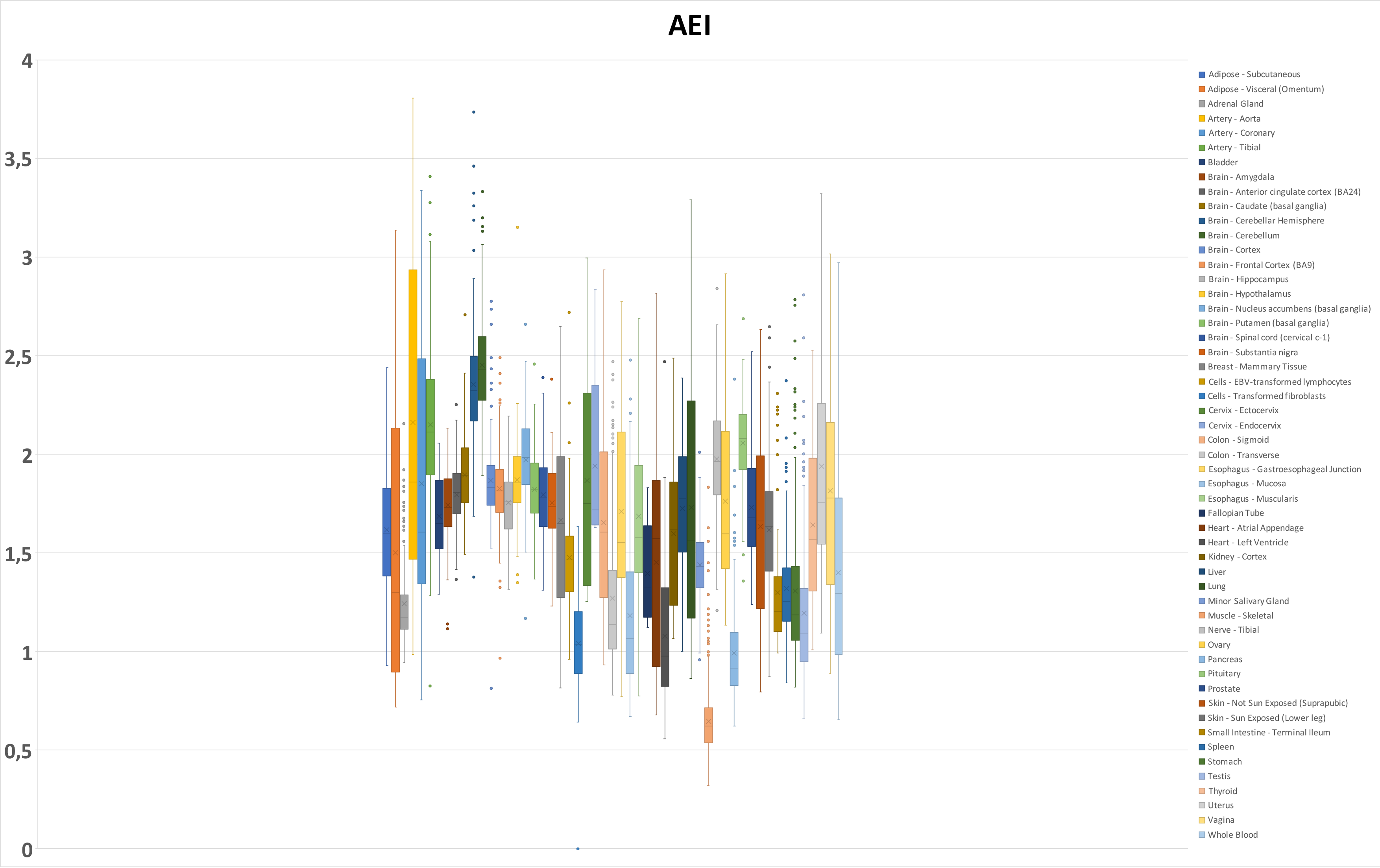


Figure S5. Distribution of REI index values across body sites.


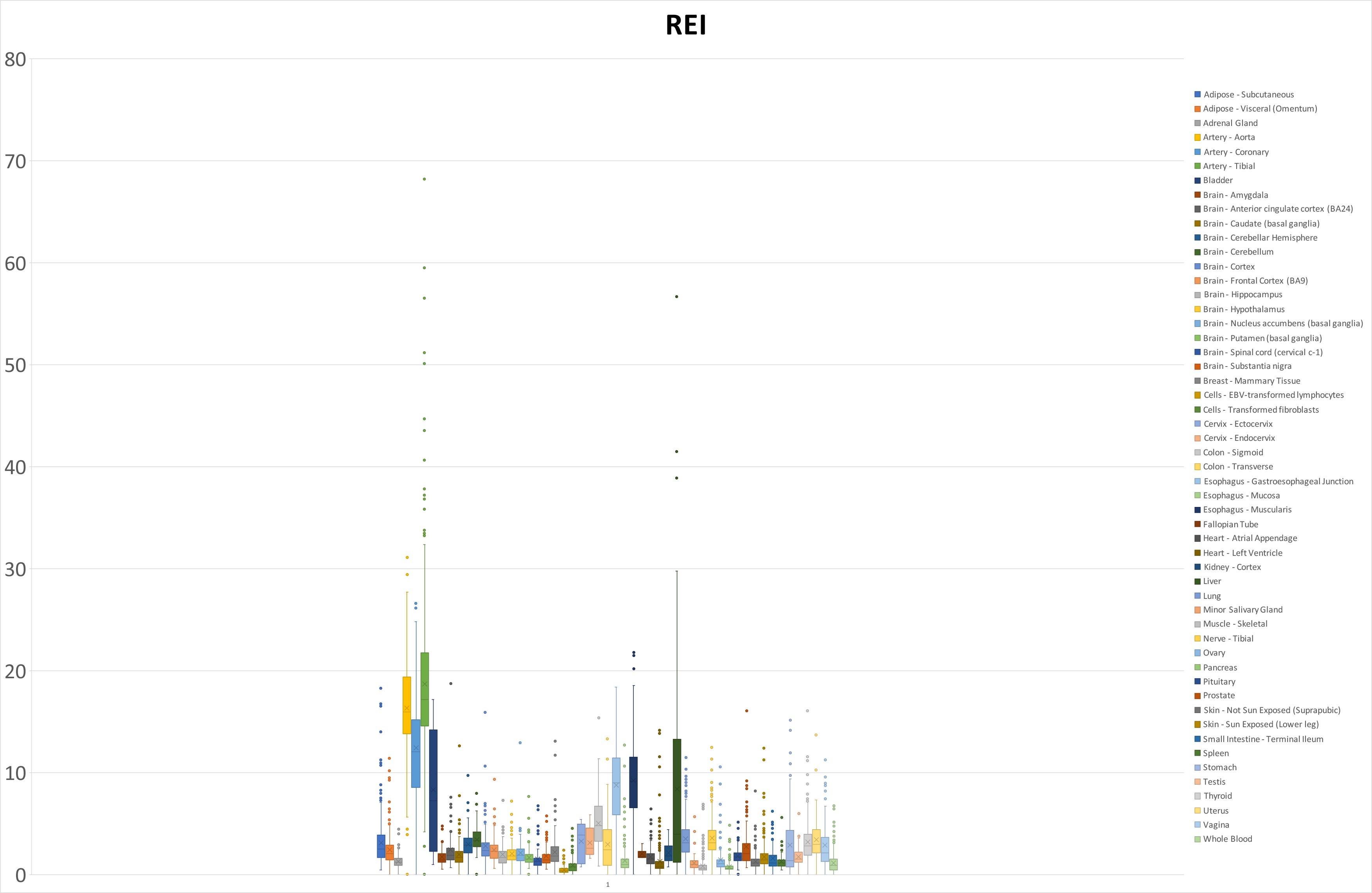

Supplement: gkaa916_Supplemental_File [file gkaa916_supplemental_file.docx]
